# High levels of serum soluble TWEAK are associated with neuroinflammation during multiple sclerosis

**DOI:** 10.1186/s12967-019-1789-3

**Published:** 2019-02-20

**Authors:** Adil Maarouf, Delphine Stephan, Marie-Pierre Ranjeva, Jean-Philippe Ranjeva, Jean Pelletier, Bertrand Audoin, Michel Khrestchatisky, Sophie Desplat-Jégo

**Affiliations:** 1 Aix-Marseille Univ, CNRS, CRMBM, Marseille, France; 2 Assistance Publique-Hôpitaux de Marseille, Hôpital de la Timone, CEMEREM, Marseille, France; 3 Assistance Publique-Hôpitaux de Marseille, Hôpital de la Timone, Pôle de Neurosciences Cliniques, Service de Neurologie, Marseille, France; 4 Aix-Marseille Université, CNRS, Faculté de Médecine, Institut de NeuroPhysiopathologie (INP), Inst Neurophysiopathol, 51 Bd P. Drammard, 13015 Marseille, France; 5 Assistance Publique-Hôpitaux de Marseille, Hôpital de la Conception, Pôle de Biologie, Service d’Immunologie, 13005 Marseille, France

**Keywords:** TWEAK, Multiple sclerosis, Neuroinflammation, Biomarker, Cytokine

## Abstract

**Background:**

Inflammation and demyelination are the main processes in multiple sclerosis. Nevertheless, to date, blood biomarkers of inflammation are lacking. TWEAK, a transmembrane protein that belongs to the TNF ligand family, has been previously identified as a potential candidate.

**Methods:**

Twenty-eight patients (9 males, 19 females) were prospectively included after a first clinical episode suggestive of multiple sclerosis and clinically followed during 3 years. Fifty-seven healthy controls were also included. TWEAK serum levels and MRI exams including magnetization transfer imaging were performed at baseline, 6- and 12-month follow-up.

**Results:**

TWEAK serum levels were significantly increased in the patient group (mean baseline = 1086 ± 493 pg/mL, mean M6 = 624 ± 302 pg/mL and mean M12 = 578 ± 245 pg/mL) compared to healthy controls (mean = 467 ± 177 pg/mL; respectively p < 0.0001, 0.01 and 0.06). Serum levels of soluble TWEAK were significantly increased during relapses, compared to time periods without any relapse (respectively 935 ± 489 pg/mL and 611 ± 292 pg/mL, p = 0.0005). Moreover, patients presenting at least one gadolinium-enhanced CNS lesion at baseline (n = 7) displayed significantly increased serum TWEAK levels in comparison with patients without any gadolinium-enhanced lesion at baseline (n = 21) (respectively 1421 ± 657 pg/mL vs 975 ± 382 pg/mL; p = 0.02). Finally, no correlation was evidenced between TWEAK serum levels and the extent of brain tissue damage assessed by magnetization transfer ratio.

**Conclusions:**

The present study showed that TWEAK serum levels are increased in MS patients, in relation to the disease activity. This simple and reproducible serum test could be used as a marker of ongoing inflammation, contributing in the follow-up and the care of MS patients. Thus, TWEAK is a promising serum marker of the best window to perform brain MRI, optimizing the disease control in patients.

## Background

Multiple sclerosis (MS) is a chronic central nervous system (CNS) disease that is characterized by demyelination, inflammation and degenerative processes leading to irreversible disability [1]. Assuming the common hypothesis that degenerative processes are mainly secondary to focal and diffuse inflammation in MS, biomarkers of ongoing inflammation are needed, particularly in the perspective of therapeutics [2]. While a dozen anti-inflammatory drugs are available to clinicians, treatment decisions remain uncertain because they are based on the concept of escalation therapy. Patients commonly start with a less effective but also a less risky drug, and escalades to a more effective drug only if inflammation remains despite treatment. Thus, the depiction of inflammation is fundamental especially considering that there is an optimal therapeutic window in MS that occurs in the first years after disease onset, where the treatment may limit irreversible disability [3, 4]. The occurrence of clinical relapses and the observation of an inflammatory activity measured by CNS magnetic resonance imaging (MRI) are commonly used to depict ongoing inflammation during MS [5]. Nevertheless, the sensitivity of relapses to detect the inflammatory processes is very low [6]. Additionally, the CNS MRI explorations cannot be as frequent as would be required [7], due to MRI accessibility and cost, time of the exam, patient comfort and potentially deleterious effect of gadolinium accumulation [8]. A blood biomarker of ongoing inflammation would be eagerly beneficial in daily MS care.

MS is associated with communication disorders between the CNS and the immune system. These disorders involve specially cytokines. Among them, the cytokine TWEAK (TNFSF12) is a transmembrane protein that belongs to the TNF (tumor necrosis factor) ligand family and whose transcripts have been found in many tissues including the brain [9]. TWEAK can be released and function as a soluble cytokine [10]. Its main receptor is Fn14, a fibroblast growth factor inducible 14 kDa protein [11]. The main sources of TWEAK protein are monocytes/macrophage family cells including microglia [12]. Based on experimental MS mouse models, TWEAK has pro-inflammatory effects during CNS inflammation. Interestingly blocking the TWEAK/Fn14 pathway during immune cell recruitment across the blood brain barrier (BBB) was protective in the same model [13, 14]. Increased permeability of the BBB is an early and critical event in the development and evolution of MS. It has been established that soluble TWEAK modulated the expression of proteins that are involved in inflammation and opening of the BBB [15]. Besides, Serafini et al. described TWEAK and Fn14 up-regulation in post-mortem MS brain sections [9]. Furthermore, this increase was related to the degree of inflammation and demyelination. The absence of TWEAK/Fn14 expression in healthy brain reinforces the idea that TWEAK/Fn 14 pathway could play a role in MS pathogeny. Additionally, a membrane TWEAK expression has been described on circulating and potentially further CNS infiltrating monocytes of MS patients and not on control monocytes suggesting that TWEAK is involved in the diapedesis of monocytes during neuroinflammation [12]. Of interest, monocytes/macrophages are known to be particularly associated with tissue injury in acute MS lesions [16–18].

Based upon these previous data, the aim of the present study was to evaluate whether serum soluble TWEAK could be a reliable biomarker of neuroinflammation in MS patients. We first determined TWEAK serum levels during a longitudinal- follow-up of MS patients started at the onset of the disease. Then we studied the relationship between TWEAK serum levels and MS course/MS inflammatory activity. Finally, we investigated the prognostic value of TWEAK serum levels for i) clinical disability and ii) CNS tissue integrity using advanced MRI techniques (magnetization transfer imaging).

## Materials and methods

### Patients and study design

Twenty-eight patients (9 males, 19 females) who attended the division of neurology of the Aix Marseille University Hospital were included in this prospective longitudinal study. For inclusion, a neurologist examined patients after a first demyelinated clinical episode suggestive of multiple sclerosis. Patients were included based on the following criteria: (i) age between 18 and 45; (ii) occurrence of the first presumed inflammatory demyelinating event in the CNS involving either the optic nerve, the spinal cord, a brain hemisphere, or the brainstem; (iii) no previous history of neurological symptoms suggestive of demyelination; (iv) no possible alternative diagnoses at the inclusion visit based on clinical examination and biological tests (especially diagnosis of systemic lupus erythematous, antiphospholipid antibody syndrome, Behcet disease, sarcoidosis, Lyme’s disease, cerebral arteritis, brain lymphoma, have been excluded); (v) fulfill at least the dissemination in space criteria according to Polman et al. [19] or presence of oligoclonal bands in the CSF analysis; (vii) first blood sample and first advanced MRI in the 12 months after the first clinical episode, not necessarily the same day as the first blood collection; (viii) no corticoids in the month before the first MRI and no previous administration of immunomodulatory or immunosuppressive drugs; (ix) no pregnancy.

Blood samples were collected thrice by venous puncture: at baseline and 6 (M6) and 12 months (M12) after the first blood collection. After blood centrifugation, serum samples were collected, aliquoted and rapidly stored at − 80 °C until analysis. At the time of blood sample collection, the serum C reactive protein concentration and the blood monocyte count were evaluated. Control blood samples were obtained from 57 healthy blood donors with no evidence of autoimmune/chronic inflammatory disease. Blood samples from healthy controls were only collected at baseline.

Patient disability was rated using the Kurtzke Expanded Disability Status Scale (EDSS) [20] at baseline, M6 and M12 on the day of the MRI exam and at M24 and M36 with a general exam.

### Quantification of soluble TWEAK

Serum concentrations of soluble TWEAK were determined in duplicates by using a commercial ELISA kit purchased from Bender Medsystems (Vienna, Austria), according to the manufacturer’s protocol and previous local experience showing a good intra and inter-assay reproducibility (CV < 10%) of this technique in serum and CSF [12]. The sensitivity of the test was 16 pg/mL. The absorbance was read within 30 min using a spectrophotometer Infinite ™ TECAN, (Mannedorf, Switzerland) at a wavelength of 450 nm.

### CNS MRI acquisition

Patients were scanned with 3T commercially available MRI systems (Verio MR system Siemens, Erlangen Germany) at baseline, M6 and M12. The protocol included transverse fast spin-echo proton density-weighted and T2-weighted sequences (TR/TE1/TE2 = 6530/8.8/88 ms, 44 contiguous sections, 3-mm section thickness, in-plane resolution 1 mm^2^), transverse proton density-weighted spoiled gradient-echo sequences (TR/TE = 750/4.5 ms, 44 contiguous sections, 3-mm section thickness, in-plane resolution 1 mm^2^) performed without (M0) and with (Mmt) magnetization transfer (MT) saturation (Gaussian shape, 1.5-kHz off-water resonance, 500°). Transverse spin-echo T1-weighted sequence (TR/TE = 500/8.4 ms, 44 contiguous sections, 3-mm section thickness, in-plane resolution 1 mm^2^) was also performed before and 5 min after intravenous administration of 0.1 mmol/kg of gadolinium (Gd) chelate to identify lesions enhanced by gadolinium. Finally, for segmentation, a sagittal 3D high-resolution MPRAGE was also acquired before administration of gadolinium (TE/TR = 3/2300 ms, TI = 900 ms, 160 slices, isotropic spatial resolution of 1 mm^3^).

### Image analysis

All conventional images were analyzed by a neurologist with more than 10 years’ experience in multiple sclerosis (AM). The visual analysis consisted of post gadolinium T1-enhanced lesion count and T2 lesion delimitation and volume estimation.

T2 lesions were delineated at baseline, M6 and M12 on the T2-weighted images by means of a semi-automated method [18] by the same experienced neurologist (AM).

Magnetization transfer ratio (MTR) maps were calculated on a voxel-by-voxel basis according to the following equation: MTR = ((M0 − Mmt)/M0), where M0 and Mmt were the images obtained respectively without and with the magnetization transfer saturation pulse (ImCalc tool, SPM12, Wellcome Trust Center for Neuroimaging, London).

T2-weighted images and T2 lesions masks were co-registered onto the Mmt images using the normalized mutual information procedure (nearest neighbour, SPM12). The co-registered masks of the T2 lesions were applied on the MTR maps to extract the MTR value of lesions.

T1-weighted images were segmented to determine for each voxel the probability of belonging to one of the 3 classes: grey matter (GM), white matter (WM) or cerebrospinal fluid (SPM12). Segmentation processing could be disrupted by the presence of focal demyelinating lesions. To prevent this pitfall, T2 lesions were first delineated at baseline, M6 and M12 onto the T2-weighted images to obtain a T2-lesion mask. Then, we used a homemade lesion filling/in-painting method, which consists to perform a first brain segmentation of T1-MPRAGE images onto three compartments: GM, WM and CSF. The corresponding co-registered lesions mask (nearest neighbour, SPM12) was used to fill T1 hypo-intensities within normal-appearing whiter matter mean values. After that step, a second brain segmentation is performed on the lesion-controlled T1-images. Finally, T1-weighted images and the GM and WM masks derived from the second segmentation are co-registered onto the Mmt images using trilinear interpolation (SPM12). These steps allow us to create a normal appearing grey matter (NAGM) and normal appearing white matter (NAWM) masks in Mmt space, obtained by removing the T2W lesion mask in the Mmt space (obtained during the step performed to extract MTR values of the lesions) from both the GM and WM masks derived from the second segmentation.

The co-registered masks of the NAGM and NAWM were applied on the MTR maps to extract the MTR values.

At the end of the pipeline, we obtained MTR values of each lesion, classified according to their enhancement and MTR values in the NAGM and NAWM.

### Statistical analysis

Two-group comparison between patients and controls were performed with a non-parametric Wilcoxon-Mann–Whitney test for quantitative variables like age or TWEAK serum levels and Fisher test for qualitative variables like gender.

Two-group comparison of TWEAK serum levels in patients according to the presence or absence of relapses or gadolinium-enhanced lesion(s) were performed with a non-parametric Wilcoxon-Mann–Whitney test.

Spearman’s rank correlation coefficient was used to test the correlation between TWEAK serum levels and C Reactive protein and monocyte count, EDSS at baseline and EDSS change, MT ratio in the three brain compartments (GM, WM and lesions).

ROC analyzes were performed to estimate the sensitivity and specificity of TWEAK serum levels to distinguish patients from controls or to distinguish active patients from non-active patients. Activity was defined by the presence of a relapse or at least one gadolinium active lesion.

Finally, a repeated measures ANOVA was performed in the patients group to assess the potential effect of age and sex in the results.

The p-values less than 0.05 were considered statistically significant.

The software used for this statistical assessment was GraphPad PRISM 7.0.a (GraphPad Software Inc., San Diego, CA, USA) and JMP 9.0.1 for the MANOVA (SAS Institute Inc).

For the MRI images, a voxel-based statistical mapping analysis was also performed in assess potential brain structural modifications correlated with serum TWEAK levels. For each subject, co-registered in the Mmt space 3D-MPRAGE images were spatially normalized into the Montreal Neurological Institute space (SPM12) and the corresponding spatial transformation was applied to the MTR maps followed by smoothing with a 8 mm FWHM Gaussian kernel. A voxel-based statistical mapping analysis (SPM12) was conducted onto the spatially normalized and smoothed MTR maps of all subjects using a one-sample t-test (p = 0.005, FDR corrected p = 0.05).

## Results

### Subjects

Twenty-eight patients were included in the present study (19 women/9 men) with a mean age of 32 ± 9.6 yo, a mean disease duration of 10.2 ± 7.5 months and a median baseline EDSS of 1 ± 1.1. No patient was treated for multiple sclerosis at baseline, 14 patients received a disease modifying therapy (DMT) at M36, among them 6 received a second line DMT. Twenty-seven out of the 28 patients responded to the McDonald 2010 criteria of MS at M36 and 16 out of the 28 patients presented a second relapse at M36 (clinically definite multiple sclerosis, [21]) (Table 1).

**Table 1:** Demographic data of population

	MS patients	Healthy controls	p value
Age (years, SD)	32 ± 9.6	38 ± 14.5	0.09
Sex (F/M)	19 F/9 M	27 F/30 M	0.11
EDSS at baseline mean (SD)	1 ± 1.1	NA	/
DMT at 3 years	14/28	NA	/
TWEAK serum levels (baseline, pg/mL)	1086 ± 493	467 ± 177	p < 0.0001

*MS* multiple sclerosis, *SD* standard deviation, *F* female; *M* male, *EDSS* Expanded Disability Status Scale; *DMT* disease modifying treatment

### TWEAK serum levels in MS

**Fig. 1:**
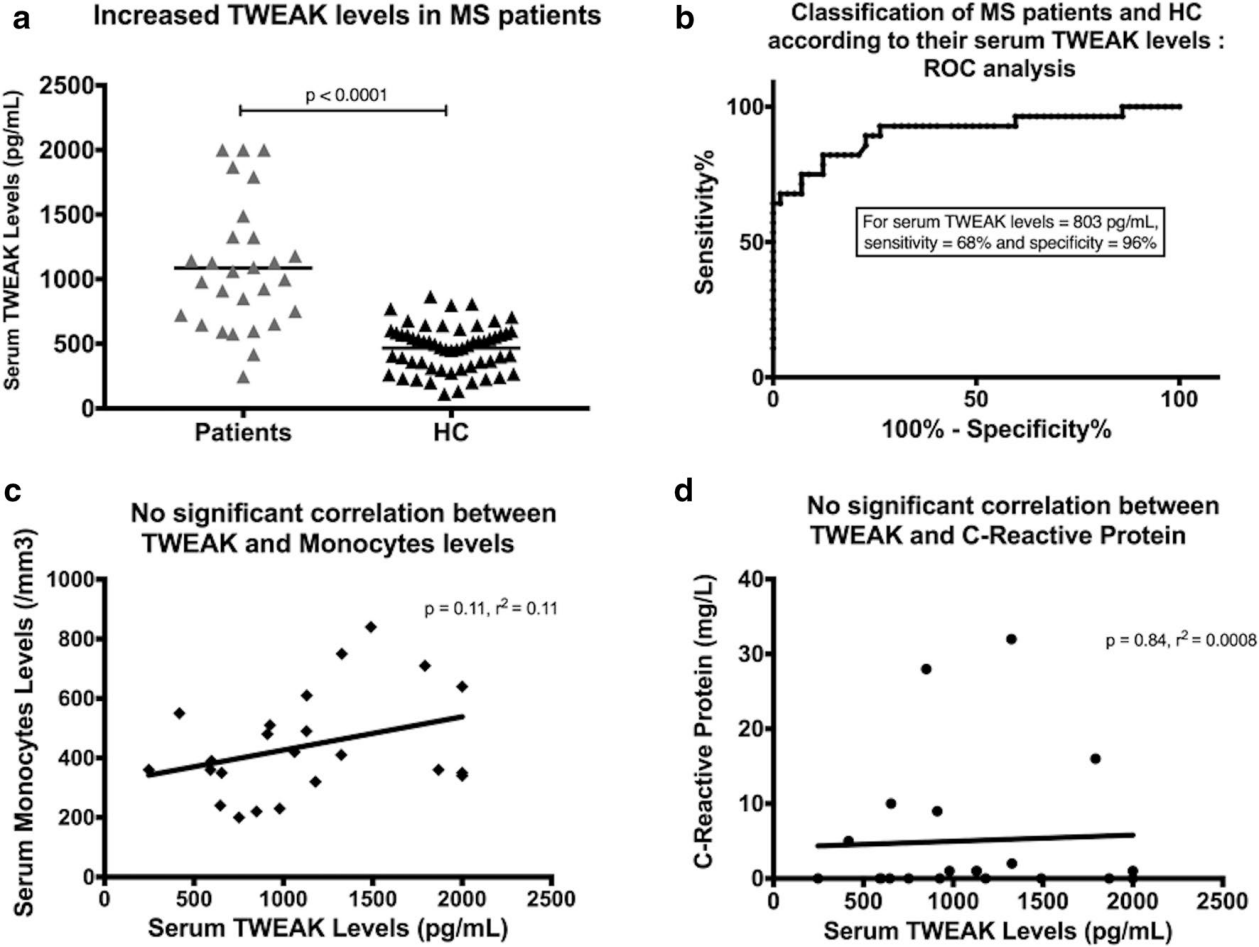
TWEAK serum levels at baseline. **a** TWEAK serum level values for each patient and each control at baseline. TWEAK serum levels were increased in the patient group (mean = 1086 ± 493 pg/mL) compared to healthy controls (mean = 467 ± 177 pg/mL; p < 0.0001); **b** ROC analysis showing a sensitivity of 68% and a specificity of 96% for TWEAK serum levels to distinguish patients from controls for a value of 803 pg/mL; Elevated TWEAK serum levels were not associated with **c** serum monocyte levels (p = 0.11) or **d** an increase of the serum C reactive protein (p = 0.84)

**Fig. 2:**
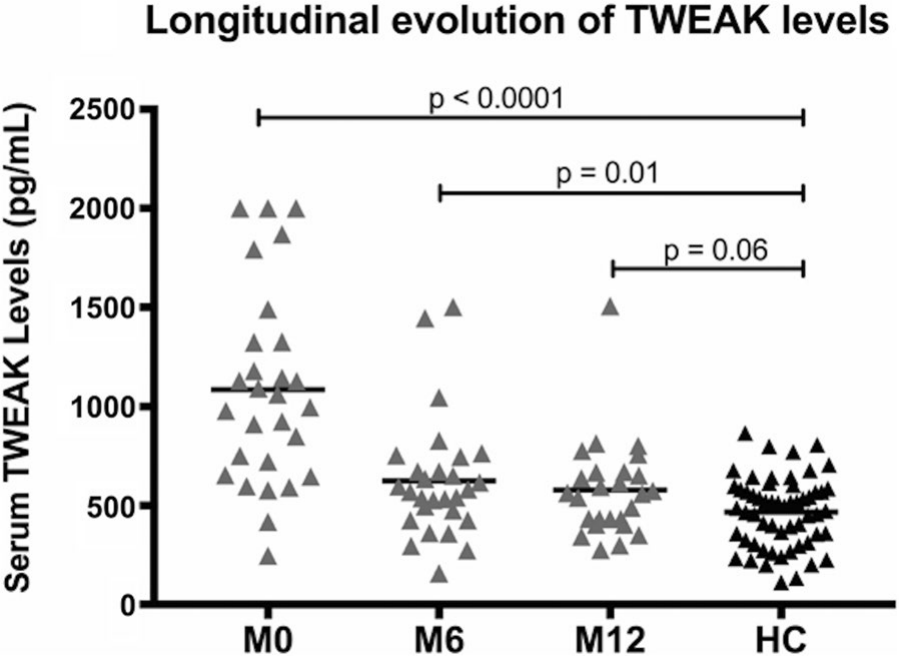
Longitudinal TWEAK serum levels. TWEAK serum level values. We note a global decrease during the first year following onset of disease, but these levels still remained higher compared to controls at 6 months (p = 0.01) and at 12 months (p = 0.06)

At baseline, TWEAK serum levels were significantly increased in the patient group (mean = 1086 ± 493 pg/mL) compared to healthy controls (mean = 467 ± 177 pg/mL; p < 0.0001) (Fig. 1). The maximum TWEAK serum level in controls was 866 pg/mL and the 98% percentile was 803 pg/mL. For this threshold, a ROC analysis showed a sensitivity of 68% and a specificity of 96% for TWEAK serum levels to distinguish patients from controls (Fig. 1). For the only one patient who did not respond to the McDonald 2010 criteria at M36, the TWEAK serum level was 911 pg/mL. Elevated TWEAK serum levels were not associated with an increase of the serum C reactive protein, biomarker of systemic inflammation observed especially during infectious diseases (p = 0.84). No correlation was observed between serum soluble TWEAK and serum monocyte levels (p = 0.11) (Fig. 1). Finally, TWEAK serum levels globally decreased during the first year following the disease onset but remained still higher compared to baseline values of TWEAK serum levels of healthy controls at 6 months (p = 0.01) and at 12 months (p = 0.06) (Fig. 2).

We performed repeated measures ANOVA in the patient group to assess the potential effect of age or sex on the results over time. Thus the age and sex did not have an effect in the patient group regarding to the follow-up (or time; respectively p = 0.61 and p = 0.51).

### TWEAK and MS disease activity

#### Relapses

**Fig. 3:**
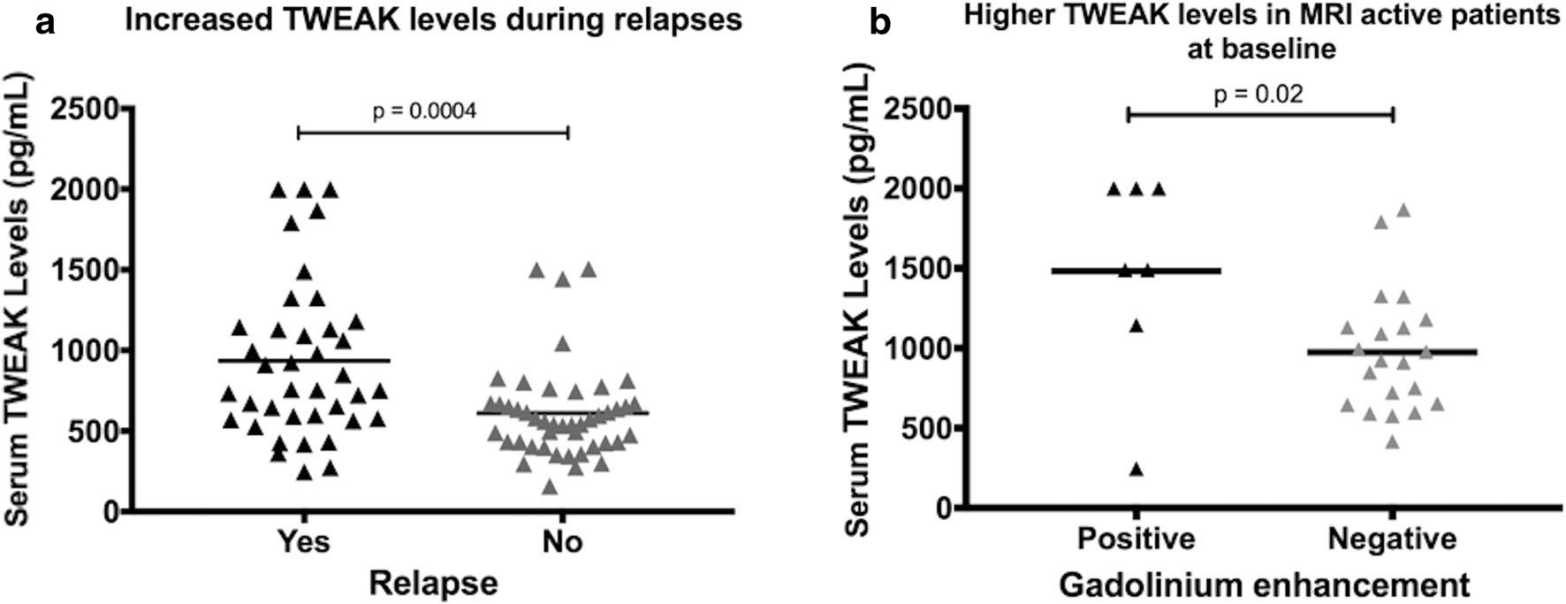
TWEAK serum levels according to presence of disease activity. **a** The serum levels of soluble TWEAK were significantly increased during relapses, compared to time periods without any relapse (respectively 935 ± 489 pg/mL and 611 ± 292 pg/mL (p = 0.0005)); **b** The same profile was shown with MRI activity at baseline with serum TWEAK levels of 1421 ± 657 pg/mL in patients who present gadolinium enhancement compared to 975 ± 382 pg/mL in case of absence of gadolinium enhancement (p = 0.02)

Thirty-nine serum samples out of 84 collected at the three time points were collected during a clinical relapse of the disease. The serum levels of soluble TWEAK were significantly increased during relapses, compared to time periods without any relapse (respectively 935 ± 489 pg/mL and 611 ± 292 pg/ml (p = 0.0005)) (Fig. 3).

#### Gadolinium enhanced lesions

Moreover, patients presenting at least one gadolinium-enhanced CNS lesion at baseline (n = 7) displayed significantly increased TWEAK serum levels in comparison with patients without any gadolinium-enhanced lesion at baseline (n = 21) (respectively 1421 ± 657 pg/mL vs 975 ± 382 pg/mL; p = 0.02) (Fig. 3).

A ROC analysis showed that a TWEAK serum level higher than 828 pg/mL had a sensitivity of 38% and a specificity of 90% to distinguish active and non-active patients.

### TWEAK and disability/tissue integrity

The mean EDSS at baseline for MS patients was estimated to 0.9 ± 1.1, and remains stable at M36. Thus, because of the stability of EDSS during the 3-year follow-up, no correlation was established between soluble TWEAK levels and baseline EDSS or EDSS variation during follow-up.

**Fig. 4:**
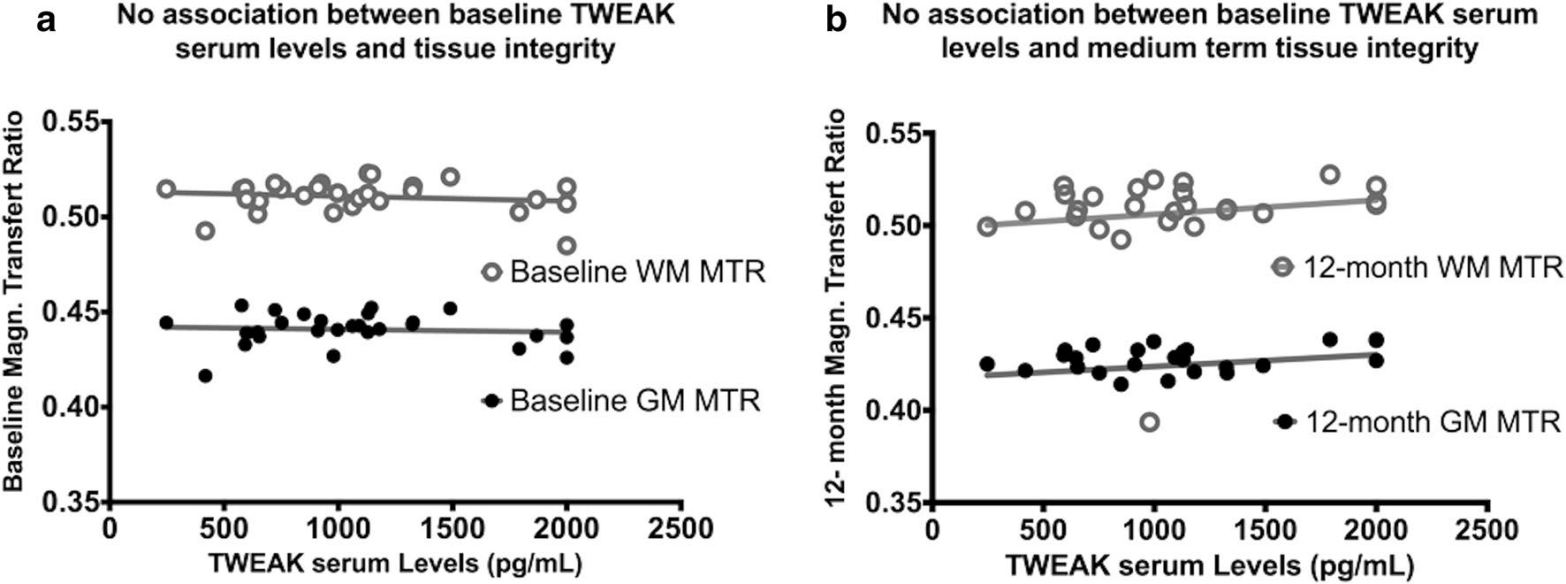
TWEAK MTR. TWEAK serum levels at baseline according to magnetization transfer ratio (MTR) at baseline (**a**) and at 1 year (**b**) in white and gray matter. No correlation was evidenced at any time and with any brain tissue

Tissue integrity was assessed by MTI, an MRI advanced technique that estimates the physical and chemical interactions between the free water proton pool and the protons close to macromolecules called the bound pool. A radiofrequency energy is applied exclusively to the bound pool leading to a decrease of signal pro rata of the amount of interactions. The healthier the tissue, the higher are the interactions between the two environments (free water pool and bound pool), the higher the signal decrease after the radiofrequency energy pulse, the higher is the MTR. In the present study, no significant correlation was found between TWEAK serum levels and MTR within and without MS lesions at baseline (p = 0.34 for lesions, p = 0.45 for normal appearing white matter and p = 0.66 for grey matter) (Fig. 4). Furthermore, at 12 months, there was still no significant correlation between TWEAK serum levels and MTR with and without MS lesions (p = 0.52 for lesions, p = 0.47 for normal appearing white matter and p = 0.44 for grey matter) (Fig. 4). We also performed a voxel-based analysis in patients between TWEAK serum levels and MTR maps. Neither at baseline nor M12, any cluster was correlated to TWEAK serum levels for the threshold of p = 0.005 FDR corrected.

### Considerations in three remarkable patients

**Fig. 5:**
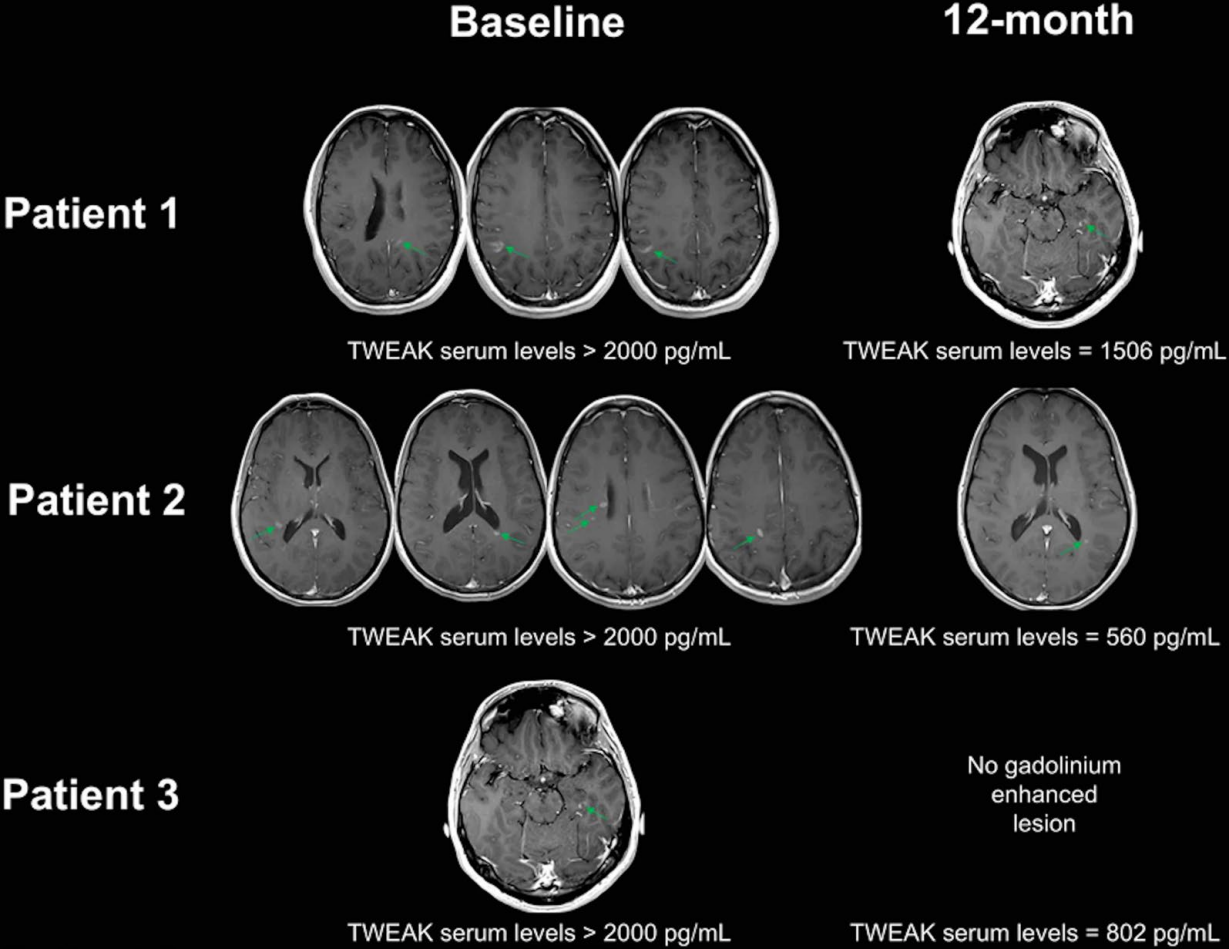
TWEAK serum levels according to gadolinium enhancement. For 3 patients, baseline TWEAK serum levels were particularly elevated and were superior to the highest standard of the TWEAK ELISA calibration curve (> 2000 pg/mL). The brain MRI (T1 after gadolinium infusion) are presented at baseline and 1 year. TWEAK serum levels at 1 year are also reported

For 3 patients, baseline TWEAK serum levels were particularly elevated and were superior to the highest standard of the TWEAK ELISA calibration curve (> 2000 pg/ml). These patients presented a relapse concomitant with the TWEAK serum level assessment and presented at least one gadolinium positive lesion at baseline. At 12 months, 2 out of these 3 patients still presented gadolinium enhanced lesions at MRI but had normal values of serum TWEAK. The third one still had increased TWEAK serum levels considering the threshold of 803 pg/mL (Fig. 5).

## Discussion

The present study brought three main findings: (i) TWEAK serum levels are increased in MS patients; (ii) this increase is related to disease activity (relapses and MRI) and (iii) no relation was evidenced between soluble TWEAK and short term brain alteration of tissue structure, suggesting that TWEAK is mainly a biomarker of inflammation in MS.

Thus, for the first time, an increase of TWEAK serum levels was shown in a homogenous population of early MS patients. All the blood samples were prospectively assessed, using the same procedure and the same ELISA kit purchased from Bender Medsystems for which the team had several years of expertise [22]. A large sample of healthy controls was also assessed to define normal biological value limits. Moreover, in the present study, we took care to control confounding factors such as systemic inflammation or peripheral monocyte proliferation independent of MS. These precautions lead to demonstrate for the first time an increase of TWEAK serum levels under inflammatory conditions in early MS, suggesting the use of TWEAK as a biomarker of inflammation in daily MS care. Nonetheless, this result was expected based on previous findings. Indeed, TWEAK exerts pleiotropic effects including apoptosis [10], angiogenesis [23, 24] but also inflammation in different cell types [15, 25–29]. In MS brain, TWEAK expression is up regulated compared to controls, particularly in tissue samples with higher inflammatory processes [9]. Macrophages, microglia and astrocytes were identified as the major sources of TWEAK in MS brain [9]. Furthermore, it was previously shown that TWEAK is expressed at the cell surface of circulating monocytes only in MS patients compared to non-MS CNS inflammatory disease controls or to patients with other neurological diseases, especially in patients included just after their first MS relapse [12]. Interestingly, none of the patients displayed TWEAK expression at the surface of lymphocytes. In experimental autoimmune encephalomyelitis (EAE), an increased severity was observed in transgenic mice overexpressing soluble TWEAK [13]. Moreover, anti-TWEAK monoclonal neutralizing antibodies have an anti-inflammatory effect in EAE and reduce disease severity [14]. All these studies strongly suggest that in MS, there is a chronic TWEAK stimulation linked to monocyte related inflammatory processes, which are particularly relevant because associated to neuro-axonal loss in MS [30, 31]. One may suppose that the increased soluble TWEAK serum levels observed in the present study are mainly due to the cleavage of membrane TWEAK from blood monocytes.

Furthermore, an originality of the present study is its longitudinal design for patients’ assessment. Evolution of TWEAK serum levels was assessed over 1 year and patients were prospectively followed over 3 years. At the group level, TWEAK serum levels decreased during follow-up but remained higher than controls. It is notable that TWEAK serum levels do not share the same kinetics at individual levels and that these levels can increase, notably when a relapse occurs. This result suggests that a TWEAK increase may be concomitant with exacerbation of inflammatory processes, and that TWEAK can be regularly measured in patient serum to advise the clinician for the best moment to perform a new MRI. Nevertheless, the kinetics and half-life of TWEAK in the serum have to be further assessed precisely to determine the optimal frequency of blood tests for monitoring MS inflammation.

The second main finding of the study is the association of high TWEAK levels with disease activity. Disease activity assessment is of primary importance in daily patient’s care, as showed, among others, by the re-examination of MS disease phenotypes [5]. In the present study, TWEAK serum levels are increased in patients presenting a relapse or a gadolinium enhanced lesion on MRI. As reported above, TWEAK expression may reflect inflammatory processes related to monocytes. Thus, an increase of TWEAK serum levels concomitant with activity in MS was expected. Moreover, a set of data strongly suggests that TWEAK may play a role in blood brain barrier (BBB) permeability modulation [15, 32–34]. The BBB is a dynamic structure composed by endothelial cells, basal lamina, astrocytes end-feet processes, pericytes and neurons. Its main function is to regulate the passage of cellular and molecular components into the CNS. BBB integrity is impaired during MS, particularly in gadolinium contrast enhanced lesions [35, 36]. It was previously showed that TWEAK has a direct effect on the interaction between astrocytes and the basal lamina, leading to changes in BBB permeability [37]. This alteration of BBB permeability seems to be induced by the pro-inflammatory effects of TWEAK by promoting secretion of cytokines [15], activating NF-KB pathways [38] and inducing the passage of inflammatory cells, particularly monocytes [39], through the BBB involving an increased expression of MMP-9 [15, 33, 38].

Nevertheless, some discrepancy between TWEAK serum levels and activity as measured in the present study can be explained by the fact that neither relapses nor gadolinium enhancement reflect all inflammatory process in MS. In fact, relapses occur only when clinically eloquent CNS regions are affected by inflammation (phenomenon 5 to 10 times less frequent than new T2 lesions occurrence on CNS MRI) [6]. Moreover, gadolinium enhancement is known to be a transient phenomenon, lasting less than 15 days [7]. Finally, TWEAK expression was showed to be particularly increased at the edges of chronic active white matter lesions [9] that are not enhanced by gadolinium [40–42]. Thus, an elevated TWEAK serum level can reflect subtle and diffuse inflammatory processes. In this regard, a future study assessing the potential value of TWEAK as a biomarker of inflammation in progressive forms of MS would be of interest, considering that these forms are known to be more affected by diffuse inflammation (microglial activation, chronic active lesions and meningeal inflammation) [43].

The last main finding of our study is that TWEAK serum levels were not related to tissue integrity at baseline and at 1 year of follow up. In the present study, tissue integrity was assessed by MTI, an MRI advanced technique that estimated the physical and chemical interactions between the free water proton pool and the protons close to macromolecules called the bound pool. This technique is widely used in imaging study in MS for two main reasons. The first is that guidelines for good quality and reproducibility of the technique were proposed in a white paper several years ago by the magnetic resonance in multiple sclerosis community (MAGNIMS) together with a group of North American experts, allowing its large use in imaging studies [44]. The second reason is that histological studies have showed in MS a robust correlation between MTR and demyelination and axonal loss [45]. The absence of correlation between TWEAK serum levels and short-term tissue damage assessed by MTI suggests that serum TWEAK up-regulation is not directly associated with demyelination and/or axonal loss.

Nevertheless, these preliminary findings need to be confirmed in large groups of MS patients including all phenotypes with a longitudinal assessment of controls. More importantly, kinetics and half-life of TWEAK in the serum should be assessed precisely and a potential correlation between TWEAK serum levels and ongoing inflammatory processes under anti-inflammatory treatment needs to be more precisely assessed. These conditions are needed for a clinical use of TWEAK as a biomarker of inflammation in clinical routine.

## Conclusion

TWEAK level in the serum of MS patients is a promising biomarker of ongoing inflammatory processes in the CNS. It is a simple and reproducible serum test that could be proposed as an accurate marker of ongoing inflammation, contributing to the follow-up and the care of MS patients. Thus, TWEAK is a promising serum marker of the best window to perform brain MRI, optimizing disease control in patients.

## Abbreviations

BBB: blood brain barrier
CNS: central nervous system
DMT: disease modifying treatment
ELISA: enzyme linked immuno sorbent assay
GM: grey matter
MS: multiple sclerosis
MPRAGE: magnetization prepared rapid gradient echo
ROC: receiver operating characteristic
MRI: magnetic resonance imaging
MTI: magnetization transfer imaging
MTR: magnetization transfer ratio
TE: time of echo
TNF: tumor necrosis factor
TI: time of inversion
TR: time of repetition
TWEAK: tumor necrosis factor-like WEAK inducer of apoptosis
WM: white matter
